# Supplementary material for: Psychological distress and coping mechanisms due to the COVID-19 pandemic among the adult population in Bo Sierra Leone. A cross-sectional study
Source: PLOS Ment Health. 2025 Feb 7;2(2):e0000177. doi: 10.1371/journal.pmen.0000177 (PMC12798522; doi:10.1371/journal.pmen.0000177)
Supplement: Attachment — Submitted filename: Response to Reviewers.docx [file pmen.0000177.s002.docx]

**Response to Reviewers**

Dear Editor

We sincerely thank the reviewers for reviewing our manuscript and providing valuable comments and suggestions. Please see our point-by-point Response to the reviewers' comments and suggestions. We have submitted a revised version of our manuscript with highlighted and tracked changes. Also, we submitted a cleaned version of our manuscript.

**Editor’s Comments**

Journal Requirements:

1. We noticed you have some minor occurrence of overlapping text with the following previous publication(s), which needs to be addressed:

- <https://doi.org/10.1371/journal.pone.0257304>
- <https://doi.org/10.21203/rs.3.rs-887072/v1>
- DOI:10.18203/2394-6040.ijcmph20205145In your revision ensure you cite all your sources (including your own works), and quote or rephrase any duplicated text outside the methods section. Further consideration is dependent on these concerns being addressed.

**Response:** we have cited all your sources, including your own works, and quote or rephrase any duplicated text outside the methods section. We ran our manuscript through plagiarism checker, and the duplicate texts detected were mainly have to do with the wordings of the instruments used in the method section. As such, we cannot change them as that will distort the original meaning of the questions asked and that will affect the internal validity of those instruments.

2. We have amended your Competing Interest statement to comply with journal style. We kindly ask that you double check the statement and let us know if anything is incorrect.

**Response:** It is correct

3. Please ensure that the Title in your manuscript file and the Title provided in your online submission form are the same.

**Response:** We have done that.

4. In the online submission form, you indicated that the datasets used and analysed during the current study are available from the corresponding author upon reasonable request. All PLOS journals now require all data underlying the findings described in their manuscript to be freely available to other researchers, either 
a. In a public repository, 
b. Within the manuscript itself, or 
c. Uploaded as supplementary information.

This policy applies to all data except where public deposition would breach compliance with the protocol approved by your research ethics board. If your data cannot be made publicly available for ethical or legal reasons (e.g., public availability would compromise patient privacy), please explain your reasons by return email and your exemption request will be escalated to the editor for approval. Your exemption request will be handled independently and will not hold up the peer review process, but will need to be resolved should your manuscript be accepted for publication. One of the Editorial team will then be in touch if there are any issues.

**Response**: We have added the following texts

*“The dataset used and analysed during the current study is available as as a supplementary file .”*

**Reviewers' comments**:

**Reviewer #1**: This is an interesting study. The manuscript provides an interesting set of findings about the psychological impact of COVID-19, which will be useful for other studies to draw upon. It can be published. However, there are some areas that could be elaborated on or improved.
**Reviewer #1 comment 1**: In the introduction, there is a statement about the potential for the impact of COVID-19 to be huge and devastating in the coming years. Could this point be elaborated on in terms of why these negative impacts are expected and in which areas?

**Response**: Thanks for your comment. We have added the following text to elaborate on what we meant in our statement. *“A lot of African economies rely on minerals and agricultural products as their primary source of foreign exchange. The global economic decline due to COVID-19 has decreased demand for these commodities and disrupted the supply chain for essential commodities, leading to scarcity and price hikes, negatively impacting most African economies [3,4]. For Instance, the United Nations Development Programme (UNDP) reported reductions in GDP growth range from -2.6% to -10.6% across ten African countries in addition to a decrease in household income, reduced government revenues and increases in extreme poverty [5]. The same UNDP report projected that indirect mortality from COVID-19 will constitute 80% of under-five mortalities in 2025 and 2030[5].”*

**Reviewer #1 comment 2**: The limitation of findings not being generalizable beyond the Bo district has been acknowledged. Nonetheless, it would be helpful to see more information on why this district was selected. If data is available, it would also be helpful to see the COVID-19 cases in the Bo district compared to other districts. A discussion of the implications of this on the findings would be useful.

**Response**: Bo district was selected because it recorded the fourth largest confirmed COVID-19 cases according to Directorate of Health Security and Emergencies Ministry of Health and Sanitation. The full data can be accessed via <https://dhse.gov.sl/> Also, it was convenient to do it in this location for economic and logistical reasons. This study was self-funded by the authors’ salary and as such it was not possible to conduct it in other districts or nationwide.

We have added the following text

*“Bo district was selected because it recorded the fourth largest confirmed COVID-19 cases, according to the Directorate of Health Security and Emergencies Ministry of Health and Sanitation, Government of Sierra Leone [42]. Western Urban (Freetown) recorded 462 cases, followed by PortLoko, Kenema and Bo districts, which had 231, 173 and 149 cases respectively [42].”*

We also acknowledged in our limitation that our finding is not representative of other districts in Sierra Leone and the whole country.

**Reviewer #1 comment 3**: In the methodology section, it would be helpful to know how the interviewees from each household were selected. Was it random sampling?
Another point to consider is whether the format of survey administration may have had any impact on the responses. It was stated that some questionnaires were self-administered while others were administered by the interviewers. Why was this the case? Could percentages for each survey administration mode be provided and could implications for interpreting the findings be stated?

**Response:** Adults from each household were selected based on availability at the time of the interview. The were not chosen using random sampling. The reason why some questionnaires were self-administered while others were administered by the interviewers was that some participants could not read and write. For that cohort, interviewer administered format was used. This cohort represents 44% of the participants recruited. We have added the following text in the limitation section

“*Fourth, 44% of participants in our study could not read and write, so an interviewer-administered format was used to collect data from this cohort. As such, there is the possibility of social desirability bias as participants reported less health impairment when interviewer-administered instruments compared to self-administered ones [70,71]. However, other studies have reported no significant difference in patient-reported outcomes when two administration formats were used [72,73].”*

Reviewer #1 comment 4: Lastly, in relation to the findings, it is stated that frontline workers had lower odds of psychological distress. However, this point is not discussed in the discussion section. This point could be elaborated on in the discussion.

**Response:** Thanks for pointing this out. We have discussed in the discussion section. The following texts have been added *“Contrary to other studies [66,67], our research suggests that frontline workers had lower odds of psychological distress. The fact that frontline workers in Sierra Leone have previously been exposed to a much more severe infectious outbreak like Ebola would have psychologically prepared them and increased their resilience [68]. A study by Tengbe et al. [69] reported that the Sierra Leonean health workers’ increased resilience during COVID-19 was due to their prior experience in providing care to patients during the 2014 Ebola outbreak.”*

**Reviewer #2:**

**Reviewer #2 Comment 1**: This paper has many strengths, but I found its underlying purpose and its contribution to the field difficult to identify. The data were collected in June/ July 2023. Vaccinations for Covid-19 began in March 2021 in Sierra Leone, and by 2023 there were no deaths from the disease (only 3 in 2022). People living in Sierra Leone have certainly experienced many challenges, but it seems that by June 2023 Covid-19 wasn't one of the most significant, so it's difficult to understand why this focus was decided upon and how it is intended to inform the mental health field.

**Response:** We understand the reviewer’s position. However, our focus here was on psychological stress response to a recent collective trauma in Sierra Leone due to COVID-19.

We agree that there were no COVID-19 cases and deaths were reported by the time of conducting the study. However, the psychological impact of COVID -19 still lingers on even after the end of the outbreak. A clear case in point was the Ebola outbreak in east and west Africa. Studies by Cenat et al 2021(ref 33) and Agaba et al 2024(ref 34) reported that the general population were psychologically affected years after the end of the outbreak. COVID-19 received unprecedented media attention in the Sierra Leone, despite low risk of transmission due to the relative low number of confirmed cases and deaths. Given that the country has experienced previous trauma due Ebola, we wanted to know to what extent has recent COVID-19 outbreak impacted the general population.

**Reviewer #2 Comment 2:** The Introduction section does not state the purpose clearly, and provides information about the Covid pandemic and response from 2021 without clarifying that this is the case. For example, the last sentence of the first para states that the impact of C-19 'is expected to be huge and devastating in the coming years', but the reference provided (the WHO covid dashboard) does not support this statement in any way.

The measures described as being introduced by the government to manage the Covid-19 situation were similarly from early on in the pandemic, and no information is provided about the situation at the time of data collection. Therefore, the study is not adequately situated in the relevant literature, and the purpose of the study is not made clear.
**Response**: We have made the necessary changes. Please see our response to Reviewer 1 comment 1. We have also added the following to properly contextualised our study

*“As the number of COVID-19 cases significantly decreased in the later stages (late 2022- early 2023), most of these measures were stopped although public health surveillance and vaccination continues [9]. As at October 2023, close two-third of the Sierra Leonean population have received at least one dose of COVID-19 vaccine [9].”*

**Reviewer #2 Comment 3:** This weakness continues in the Discussion section. Whilst each individual finding is discussed in relation to relevant literature, the overall purpose and learnings from the study are unclear. The section on 'policy practice and implementation' could usefully be expanded upon.

**Response**: We revised our policy, practice and research implications. The following texts have been added

*“Mental health needs of the Sierra Leonean population are high due to psychological impact of the civil war, recent Ebola and flooding and mudslide. However, such high need is mismatched by inadequate provision of mental health services [60]. The country currently has only one psychiatric hospital in the capital city, Freetown with limited workforce of two psychiatrists and nineteen mental health nurses [74]. Given the above findings, it is important that the Bo district health management team should increase equitable access to mental health services through adequate investment and integrating mental health into primary healthcare. Also, it is important that culturally appropriate mental health interventions need to be designed to meet the needs of the people. In addition, the training of healthcare providers in assessing and implementing mental health techniques is essential. The design of community mental health interventions -a way to integrate mental in in primary care is important to support people in their communities. Peer support programs have been shown to be effective community-based intervention as it provides for people to share their experiences and receive support from others who have gone through similar challenges [75]. To achieve this, multi-sectoral, multi-level coordinated effort from internal and external actors is required. Given that our study is cross-sectional in nature, it would be good to conduct longitudinal studies to monitor long-term mental health impacts of the COVID-19 to inform new or existing interventions..”*

**Reviewer #2 Comment 4:** The data collection, analysis and reporting appears adequate (although I'm not in a position to assess the accuracy of the sampling calculations or the regression analysis). However, the claims made for the meaning of the findings go far beyond what can be concluded from the information available (e.g. that the coping mechanisms identified could be related to challenging work environments, limited access to medications, under-reporting of mental health issues, economic impact of lockdown - there is no evidence to suggest that it is due to any of these factors).
The paper has some potential but would need to be significantly revised to focus more tightly on a clear set of aims and to ground conclusions more solidly in the data.

**Response:** Thanks for pointing this out. We have addressed the issue regarding possible explanation or reasons that explain the low coping mechanism shown by adult Sierra Leoneans in Bo district during COVID-19. Please see the text below

*“The findings from our study revealed that half of participants exhibited low coping mechanisms among participants. This could be attributed to past trauma due to the eleven years old civil war and poverty which impacted people mental health. Thus, the COVID-19 pandemic may have brought back trauma-related symptoms making it difficult for people to cope [55,56]. Also, lockdown and restrictions leading to economic hardship and fear of infection are sources of stress, which negatively impact people’s mental health and their ability to cope with everyday life [57].”*

We hope we have adequately addressed the editor and reviewers’ comments

Yours Sincerely.

Augustus Orsborne

Corresponding author
